# Life in Hot Carbon Monoxide: The Complete Genome Sequence of *Carboxydothermus hydrogenoformans* Z-2901

**DOI:** 10.1371/journal.pgen.0010065

**Published:** 2005-11-25

**Authors:** Martin Wu, Qinghu Ren, A. Scott Durkin, Sean C Daugherty, Lauren M Brinkac, Robert J Dodson, Ramana Madupu, Steven A Sullivan, James F Kolonay, William C Nelson, Luke J Tallon, Kristine M Jones, Luke E Ulrich, Juan M Gonzalez, Igor B Zhulin, Frank T Robb, Jonathan A Eisen

**Affiliations:** 1 The Institute for Genomic Research, Rockville, Maryland, United States of America; 2 Center for Bioinformatics and Computational Biology, School of Biology, Georgia Institute of Technology, Atlanta, Georgia, United States of America; 3 Center of Marine Biotechnology, University of Maryland Biotechnology Institute, Baltimore, Maryland, United States of America; 4 Johns Hopkins University, Baltimore, Maryland, United States of America; Joint Genome Institute, United States of America

## Abstract

We report here the sequencing and analysis of the genome of the thermophilic bacterium *Carboxydothermus hydrogenoformans* Z-2901. This species is a model for studies of hydrogenogens, which are diverse bacteria and archaea that grow anaerobically utilizing carbon monoxide (CO) as their sole carbon source and water as an electron acceptor, producing carbon dioxide and hydrogen as waste products. Organisms that make use of CO do so through carbon monoxide dehydrogenase complexes. Remarkably, analysis of the genome of *C. hydrogenoformans* reveals the presence of at least five highly differentiated anaerobic carbon monoxide dehydrogenase complexes, which may in part explain how this species is able to grow so much more rapidly on CO than many other species. Analysis of the genome also has provided many general insights into the metabolism of this organism which should make it easier to use it as a source of biologically produced hydrogen gas. One surprising finding is the presence of many genes previously found only in sporulating species in the Firmicutes Phylum. Although this species is also a Firmicutes, it was not known to sporulate previously. Here we show that it does sporulate and because it is missing many of the genes involved in sporulation in other species, this organism may serve as a “minimal” model for sporulation studies. In addition, using phylogenetic profile analysis, we have identified many uncharacterized gene families found in all known sporulating Firmicutes, but not in any non-sporulating bacteria, including a sigma factor not known to be involved in sporulation previously.

## Synopsis

*Carboxydothermus hydrogenoformans,* a bacterium isolated from a Russian hotspring, is studied for three major reasons: it grows at very high temperature, it lives almost entirely on a diet of carbon monoxide (CO), and it converts water to hydrogen gas as part of its metabolism. Understanding this organism's unique biology gets a boost from the decoding of its genome, reported in this issue of *PLoS Genetics*. For example, genome analysis reveals that it encodes five different forms of the protein machine carbon monoxide dehydrogenase (CODH). Most species have no CODH and even species that utilize CO usually have only one or two. The five CODH in *C. hydrogenoformans* likely allow it to both use CO for diverse cellular processes and out-compete for it when it is limiting. The genome sequence also led the researchers to experimentally document new aspects of this species' biology including the ability to form spores. The researchers then used comparative genomic analysis to identify conserved genes found in all spore-forming species, including *Bacillus anthracis,* and not in any other species. Finally, the genome sequence and analysis reported here will aid in those trying to develop this and other species into systems to biologically produce hydrogen gas from water.

## Introduction

Carbon monoxide (CO) is best known as a potent human poison, binding very strongly and almost irreversibly to the iron core of hemoglobin. Despite its deleterious effects on many species, it is also the basis for many food chains, especially in hydrothermal environments such as the deep sea, hot springs, and volcanoes. In these environments, CO is a common potential carbon source, as it is produced both by partial oxidation of organic matter as well as by multiple microbial strains (e.g., methanogens). It is most readily available in areas in which oxygen concentrations are low, since oxidation of CO will convert it to CO_2_. In hydrothermal environments, CO use as a primary carbon source is dominated by the hydrogenogens, which are anaerobic, thermophilic bacteria or archaea that carry out CO oxidation using water as an electron acceptor [1]. This leads to the production of CO_2_ and H_2_. The H_2_ is frequently lost to the environment and the CO_2_ is used in carbon fixation pathways for the production of biomass. Hydrogenogens have attracted significant biotechnological interest because of the possibility they could be used in the biological production of hydrogen gas.

Hydrogenogens are found in diverse volcanic environments [2–7]. The phylogenetic types differ somewhat depending on the environments and include representatives of bacteria and archaea. *Carboxydothermus hydrogenoformans* is a hydrogenogen that was isolated from a hot spring in Kunashir Island, Russia [2]. It is a member of the Firmicutes Phylum (also known as low GC Gram-positives) and grows optimally at 78 °C. This species has been considered an unusual hydrogenogen, in part because unlike most of the other hydrogenogens, it was believed to be strictly dependent on CO for growth. The other species were found to grow poorly unless CO was supplemented with organic substrates. Thus it was selected for genome sequencing as a potential model obligate CO autotroph.

Surprisingly, initial analysis of the unpublished genome sequence data led to the discovery that this species is not an obligate CO autotroph [8]. We report here a detailed analysis of the genome sequence of *C. hydrogenoformans* strain Z-2901, the type strain of the species, hereafter referred to simply as *C. hydrogenoformans*.

## Results/Discussion

### Genome Structure

**Figure 1:**
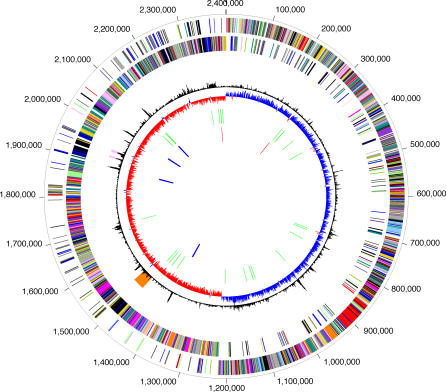
Genomic Organization of *C. hydrogenoformans* From the outside inward the circles show: (1, 2) predicted protein-coding regions on the plus and minus strands (colors were assigned according to the color code of functional classes; (3) prophage (orange) and CRISPR (pink) regions; (4) χ^2^-square score of tri-nucleotide composition; (5) GC skew (blue indicates a positive value and red a negative value); (6) tRNAs (green); (7) rRNAs (blue) and structural RNAs (red).

The *C. hydrogenoformans* genome is a single circular chromosome of 2,401,892 base pairs (bp) with a G+C content of 42.0% (Figure 1, Table 1). Annotation of the genome reveals 2,646 putative protein coding genes (CDSs), of which 1,512 can be assigned a putative function. The chromosome displays two clear GC skew transitions that likely correspond to the DNA replication origin and terminus (Figure 1). Overall, 3.0 % of the genome is made up of repetitive DNA sequences. Included in this repetitive DNA are two large-clustered, regularly interspaced short palindromic repeats (CRISPR, 3.9 and 5.6 kilobases, respectively). Each cluster contains 59 and 84 partially palindromic repeats of 30 bps, respectively (GTTTCAATCCCAGA[A/T]TGGTTCGATTAAAAC). Most repeats within each cluster are identical but they differ for one nucleotide in the middle between clusters. Repeats at ends of the smaller cluster degenerate to some extent. These types of repeats are widespread in diverse groups of bacteria and archaea [9]. The first one-third of the repeat sequence is generally conserved. Although the precise functions of these repeats are unknown, some evidence suggests they are involved in chromosome partitioning [10,11]. In addition, experiments in the thermophilic archaea *Sulfolobus solfataricus* have identified a genus-specific protein binding specifically to the repeats present in that species' genome [11].

**Table 1:**
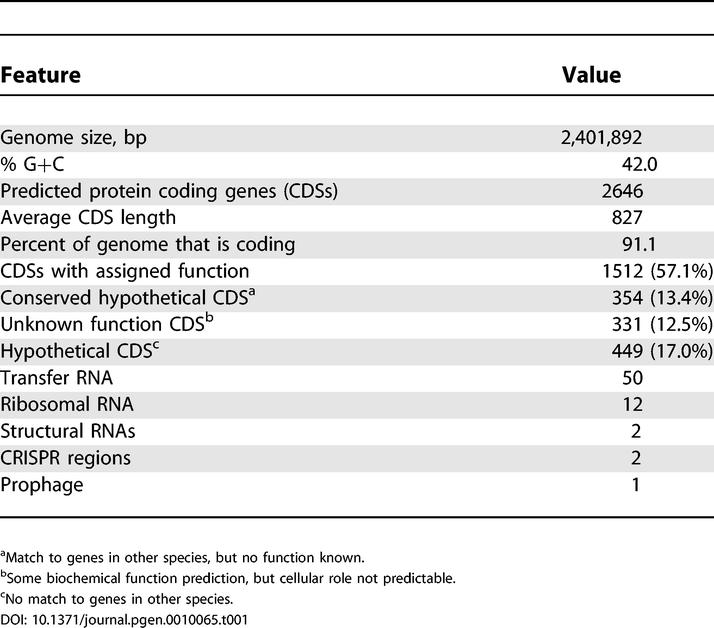
General Features of the *C. hydrogenoformans* Genome

One 35-kilobase lambda-like prophage containing 50 CDSs was identified in the genome. It is flanked on one side by a tRNA suggesting this may have served as a site of insertion. Phylogenetic analysis showed this phage is most closely related to phages found in other Firmicutes, particularly the SPP1 phage infecting *Bacillus subtilis.*

As with other members of the Phylum Firmicutes, the directions of leading strand DNA replication and transcription are highly correlated, with 87% of genes located on the leading strand. This gene distribution bias is also highly correlated with the presence of a Firmicutes-specific DNA polymerase PolC in the genome [12]. In *B. subtilis,* PolC synthesizes the leading strand, and another distinct DNA polymerase, DnaE, replicates the lagging strand [13]. In other non-Firmicutes bacteria, DnaE replicates both strands. The asymmetric replication forks of Firmicutes were proposed to contribute to the asymmetry of their gene distributions [12]. One copy of PolC and two copies of DnaE have been identified in *C. hydrogenoformans* genome. At least some of the gene distribution bias can be caused by selection to avoid collision of the RNA and DNA polymerases as well [14,15]. Despite this apparent selection, the lack of significantly conserved gene order across Firmicutes indicates that genome rearrangements still occur at a reasonably high rate.

### Phylogeny and Taxonomy

**Figure 2:**
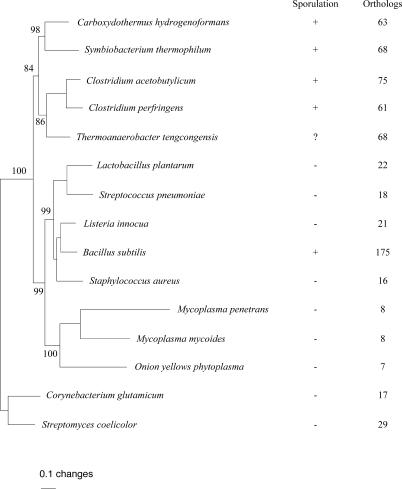
Genome Tree of Representatives of Firmicutes A maximum likelihood tree was built from concatenated protein sequences of 31 universal housekeeping genes and rooted by two outgroup Actinobacteria (high GC Gram-positives) species: *Corynebacterium glutamicum* and *Streptomyces coelicolor.* Bootstrap support values (out of 100 runs) for branches of interest are shown beside them. Each species' ability to sporulate and its number of putative orthologs of the 175 known *B. subtilis* sporulation genes are also shown.

Analysis of the complete genome of *C. hydrogenoformans* suggests that the taxonomy of this species, as well as some other organisms, needs to be revised. More specifically, phylogenetic analysis based on concatenation of a few dozen markers (Figure 2) reveals a variety of conflicts between the organismal phylogeny and the classification of some of the Firmicutes. For example, *C. hydrogenoformans* is currently considered to be a member of the Family Peptococcaceae in the Order Clostridiales [16]. Thus it should form a clade with the *Clostridium* spp. to the exclusion of other taxa for which genomes are available (e.g., *Thermoanaerobacter tengcongensis,* which is considered to be a member of Thermoanaerobacteriales). The tree, however, indicates that this is not the case and that *T. tengcongensis* and the *Clostridia* spp. are more closely related to each other than either is to *C. hydrogenoformans.* Thus we believe *C. hydrogenoformans* should be placed in a separate Order from Clostridiales.

Perhaps more surprisingly, the concatenated genome tree shows *C. hydrogenoformans* grouping with *Symbiobacterium thermophilum. S. thermophilum* is a strictly symbiotic thermophile isolated from compost and is currently classified in the Actinobacteria (also known as high GC Gram-positives) based on analysis of its 16s rRNA sequence [17]. The grouping with Firmicutes is supported by the overall level of similarity of its proteome to other species [18]. We therefore believe the rRNA-based classification is incorrect and that *S. thermophilum* should be transferred to the Firmicutes. Such inaccuracies of the rRNA trees are relatively uncommon and may in this case be due to the mixing of thermophilic and non-thermophilic species into one group. This can cause artifacts when using rRNA genes for phylogenetic reconstruction since the G+C content of rDNA is strongly correlated to optimal growth temperature.

### CO Dehydrogenases and Life in CO

**Figure 3:**
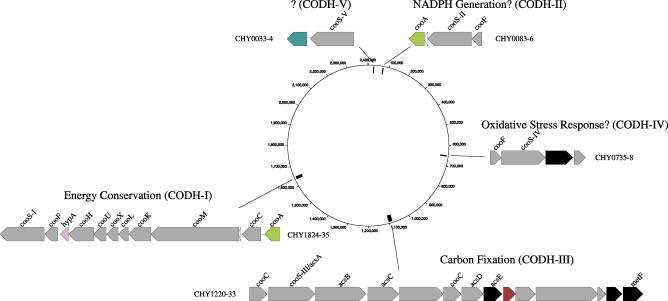
Genome Locations of Genes Predicted to Encode Five CODH Complexes The genome locations of the genes encoding the five CooS homologs (labelled CooS I-V) are shown. Also shown are neighboring genes that are predicted to encode the five distinct CODH complexes (CODH I-V) with each CooS homolog. Possible cellular roles for four of the five CODH complexes are indicated.

Anaerobic species that make use of CO do so using nickel-iron CO dehydrogenase (CODH) complexes [19,20]. These enzymes all appear to catalyze the anaerobic interconversion of CO and CO_2_. However, they vary greatly in the cellular role of this conversion and in the exact structure of the complex [19]. Analysis of the genome reveals the presence of five genes encoding homologs of CooS, the catalytic subunit of anaerobic CODHs. These five CooS encoding genes are scattered around the genome, and analysis of genome context, gene phylogeny, and experimental studies in this and other CO-utilizing species suggests they are subunits of five distinct CODH complexes, which we refer to as CODH I-V (Figure 3). The CooS homologs are named accordingly. Specific details about each complex and proposed physiological roles are given in the following paragraphs.

### Energy Conservation (CODH-I)

**Figure 4:**
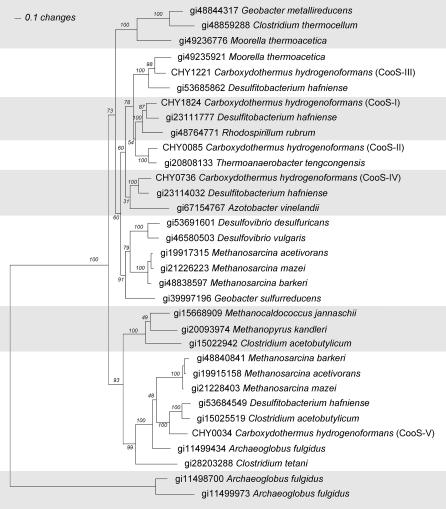
Phylogenetic Tree of CooS Homologs The figure shows a maximum-likelihood tree of CooS homologs. The tree indicates the five CooS homologs in *C. hydrogenoformans* are not the result of recent duplications but instead are from distinct subfamilies. The other CooS homologs included in the tree were obtained from the NCBI nr database and include some from incomplete genome sequences generated by United States Department of Energy Joint Genome Institute (http://www.jgi.doe.gov/).

A catalytic subunit (CooS-I, CHY1824) and an electron transfer protein (CooF, CHY1825) of CODH are encoded immediately downstream of a hydrogenase gene cluster (*cooMKLXUH,* CHY1832–27) that is closely related to the one found in *Rhodospirillum rubrum* [21]. These eight proteins form a tight membrane-bound enzyme complex that converts CO to CO_2_ and H_2_ in vitro [1,22]. In *R. rubrum,* this CODH/hydrogenase complex was proposed to be the site of CO-driven proton respiration where energy is conserved in the form of a proton gradient generated across the cell membrane [21]. Based on the high similarities in protein sequences and their gene organization, this set of genes were suggested to play a similar role in energy conservation in *C. hydrogenoformans* [1]. Consistent with this, this *cooS* gene is in the same subfamily as that from *R. rubrum* (Figure 4).

### Carbon Fixation (CODH-III)

**Figure 5:**
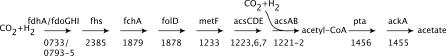
Predicted Complete Acetyl-CoA Pathway of Carbon Fixation in *C. hydrogenoformans* Genes predicted to encode each step in the acetyl-CoA pathway of carbon fixation were identified in the genome. The locus numbers are indicated on the figure.

Anaerobic bacteria and archaea, such as methanogens and acetogens, can fix CO or CO_2_ using the acetyl-CoA pathway (also termed the Wood-Ljungdahl pathway), where two molecules of CO_2_, through a few steps, are condensed into one acetyl-CoA, a key building block for cellular biosynthesis and an important source of ATP [23]. The key enzyme of the final step (a CODH/acetyl-CoA synthase complex) has been purified from *C. hydrogenoformans* (strain DSM 6008) cultured under limited CO supply and shown to be functional in vitro [24]. Genes encoding this complex and other proteins predicted to be in this pathway are clustered in the genome (CHY1221–7). This cluster is very similar to the *acs* operon from the acetogen *Moorella thermoacetica* which encodes the acetyl-CoA pathway machinery [25]. The phylogenetic tree also shows that CooS-III is in the same subfamily as the corresponding gene in the *M. thermoacetica acs* operon (Figure 4), suggesting they have the same biological functions. In addition, all the genes in the acetyl-CoA pathway have been identified in the *C. hydrogenoformans* genome and activities of some of those gene products have been detected (Figure 5), prompting us to propose that this organism carries out autotrophic fixation of CO through this pathway. This is consistent with the observation that key enzymes for the other known CO_2_ fixation pathways, such as the Calvin cycle, the reverse tricarboxylic acid cycle, and 3-hydroxypropionate cycle are apparently not encoded in the genome.

### Oxidative Stress Response (CODH-IV)

*C. hydrogenoformans,* though an anaerobe, has to deal with oxidative challenges present in the environment from time to time. Unlike aerobes, many anaerobes are proposed to use an alternative oxidative stress protection mechanism that depends on proteins such as rubrerythrin [26,27]. With few exceptions, rubrerythrin-like proteins have been found in complete genomes of all anaerobic and microaerophilic microbes but are absent in aerobic microbes [28]. Rubrerythrin is thought to play a role in the detoxification of reactive oxygen species by reducing the intermediate hydrogen peroxide, although the exact details remain elusive [28,29]. *C. hydrogenoformans* encodes three rubrerythrin homologs. One of them forms an operon with genes encoding CooS-IV, a CooF homolog, and a NAD/FAD-dependent oxidoreductase (CHY0735–8, Figure 3), suggesting that their functions are related. Here we speculate that this operon encodes a multi-subunit complex where electrons stripped from CO by the CODH are passed to rubrerythrin to reduce hydrogen peroxide to water, with CooF and the NAD/FAD-dependent oxidoreductase acting as the intermediate electron carriers. Therefore, CODH-IV may play an important role in oxidative stress response by providing the ultimate source of reductants.

### Others

Two other homologs of CooS are encoded in the genome. The gene encoding CooS-II (CHY0085) was originally cloned with the neighboring *cooF* (CHY0086) [30] and the complex was purified as functional homodimers [1]. This complex (CODH-II) is membrane-associated and an in vitro study showed it might have an anabolic function of generating NADPH [1]. Its structure has been solved [31]. The role of CooS-V (CHY0034) is more intriguing as it is the most deeply branched of the CooSs (Figure 4) and is not flanked by any genes with obvious roles in CO-related processes.

Aerobic bacteria metabolize CO using drastically different CODHs that are unrelated to the anaerobic ones. The CODHs from aerobes are dimers of heterotrimers composed of a molybdoprotein (CoxL), a flavoprotein (CoxM), and an iron-sulfur protein (CoxS) and belong to a large family of molybdenum hydroxylases including aldehyde oxidoreductases and xanthine dehydrogenases [32]. These enzymes characteristically demonstrate high affinity for CO, and the oxidation is typically coupled to CO_2_ fixation via the reductive pentose phosphate cycle.

*C. hydrogenoformans* has one gene cluster (CHY0690–2) homologous to the *coxMSL* cluster in *Oligotropha carboxidovorans,* the most well-studied aerobic CODHs. However, our phylogenetic analysis showed that the *C. hydrogenoformans* homolog of CoxL does not group within the CODH subfamily. Therefore, we conclude that it is unlikely that this gene cluster in *C. hydrogenoformans* encodes a CODH, although that needs to be tested. Of the available published and unpublished genomes, only *R. rubrum* appears to have both an anaerobic CODH and a close relative of the aerobic *O. carboxidovorans* CODH. Accordingly, *R. rubrum,* a photosynthetic bacterium, can grow in the dark both aerobically and anaerobically using CO as an energy source.

Structures of both the Mo- and Ni-containing enzymes have been published recently. The crystal structure of CooS-II from *C. hydrogenoformans* is a dimeric enzyme with dual Ni-containing reaction centers each connected to the enzyme surface by 70-Å hydrophobic channels through which CO transits [31]. This channeling, also confirmed experimentally [33,34], explains the mechanism of CO use as a central metabolic intermediate despite its low solubility and generally low concentration in geothermal environments.

### Sporulation

**Figure 6:**
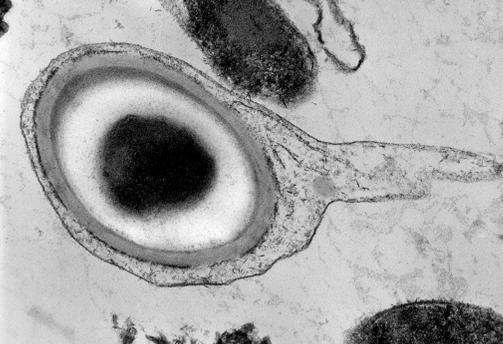
An Electron Micrograph of a *C. hydrogenoformans* Endospore The finding of homologs of many genes involved in sporulation in other species led us to test whether *C. hydrogenoformans* also could form an endospore. Under stressful growth conditions, endospore-like structures form. We note that even though homologs could not be found in the genome for many genes that in other species are involved in protective outer-layer (cortex, coat, and exosporium) formation, those structures seem to be visible and intact.

The *C. hydrogenoformans* genome encodes a large number of homologs of genes involved in sporulation in other Firmicutes, spanning all stages of sporulation (Table 2). Among those are the master switch gene *spo0A* and all sporulation-specific sigma factors, σ^H^, σ^E^, σ^F^, σ^G^, and σ^K^. However, sporulation has not been previously reported for this species. With this in mind, we set out to re-examine the morphology of *C. hydrogenoformans* cells and found endospore-like structures when cultures were stressed (Figure 6).

**Table 2:**
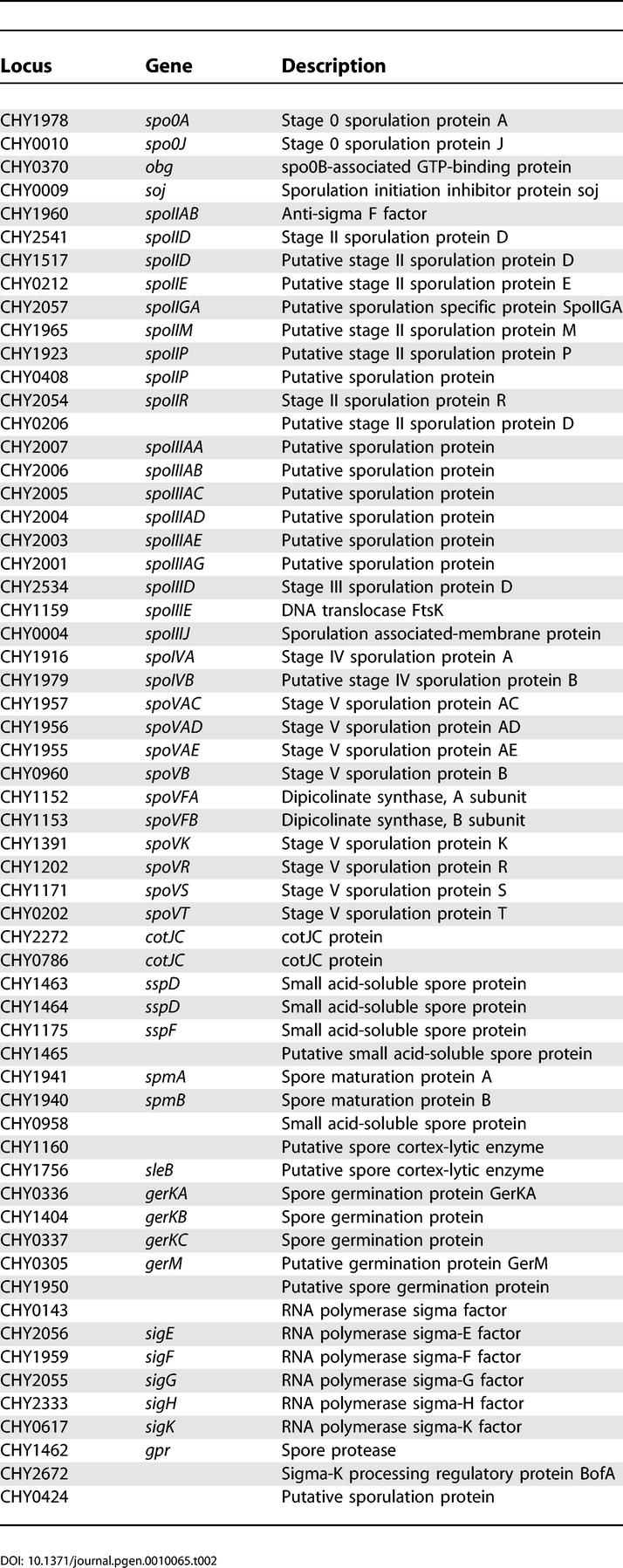
Orthologs of Known *Bacillus subtilis* Sporulation Genes in *C. hydrogenoformans*

**Figure 7:**
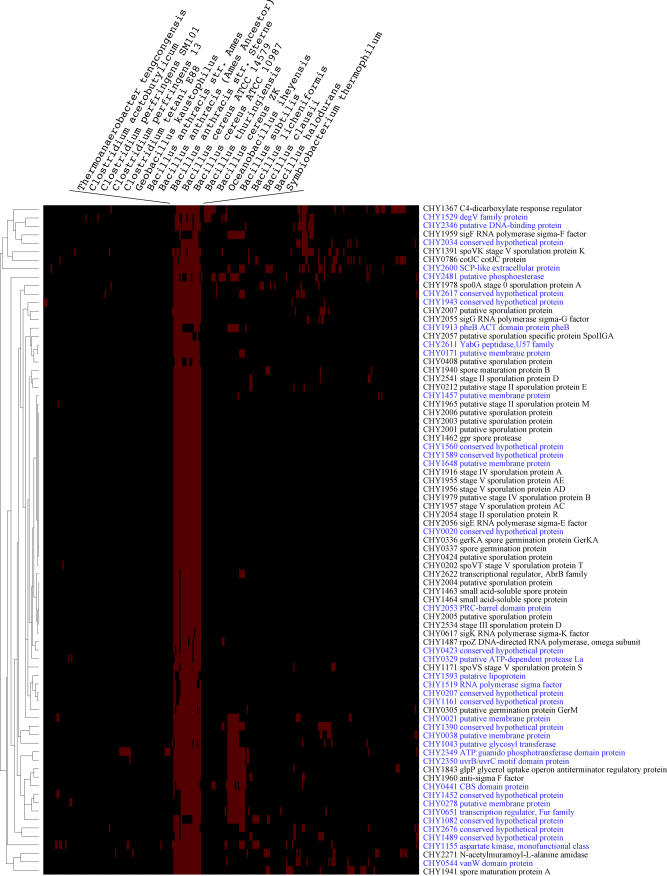
Phylogenetic Profile Analysis of Sporulation in *C. hydrogenoformans* For each protein encoded by the *C. hydrogenoformans* genome, a profile was created of the presence or absence of orthologs of that protein in the predicted proteomes of all other complete genome sequences. Proteins were then clustered by the similarity of their profiles, thus allowing the grouping of proteins by their distribution patterns across species. Examination of the groupings showed one cluster consisting of mostly homologs of sporulation proteins. This cluster is shown with *C. hydrogenoformans* proteins in rows (and the prediced function and protein ID indicated on the right) and other species in columns with presence of a ortholog indicated in red and absence in black. The tree to the left represents the portion of the cluster diagram for these proteins. Note that most of these proteins are found only in a few species represented in red columns near the center of the diagram. The species corresponding to these columns are indicated. We also note that though most of the proteins in this cluster, for which functions can be predicted, are predicted to be involved in sporulation and some have no predictable functions (highlighted in blue). This indicates that functions of these proteins' homologs have not been characterized in other species. Since these proteins show similar distribution patterns to so many proteins with roles in sporulation, we predict that they represent novel sporulation functions.

We then used phylogenetic profile analysis to look for other possible sporulation genes in the genome. Phylogenetic profiling works by grouping genes according to their distribution patterns in different species [35]. Proteins that function in the same pathways or structural complexes frequently have correlated distribution patterns. Phylogenetic profile analysis identified an additional set of 37 potential sporulation-related genes (Figure 7). Those genes are generally *Bacillales-* and *Clostridiales-*specific, consistent with the fact that endospores have so far only been found in these and other closely related Firmicutes. Most of the novel genes are conserved hypothetical proteins, whereas a few are putative membrane proteins. In support, a few of those novel sporulation genes have been shown to be involved in *Bacillus subtilis* sporulation by experimental studies [36,37]. The rest of the genes are thus excellent candidates for encoding known sporulation functions that have not been assigned to genes or previously unknown sporulation activities. Strikingly, within this group of genes, in addition to other known sporulation-specific sigma factors (σ^E^, σ^F^, σ^G^, and σ^K^), we identified a sigma factor (CHY1519) that was not known to be associated with sporulation previously. σ^I^, its putative ortholog in *B. subtilis,* has shown some association with heat shock [38]. It remains to be determined experimentally whether this sigma factor is involved in sporulation, and if so, the regulatory network it controls.

A search of known sporulation-related genes in *B. subtilis* against *C. hydrogenoformans* revealed that many of them are missing in the genome. Of the 175 *B. subtilis* sporulation-related genes we compiled from the genome annotation and literature [39,40], half have no detectable homologs in *C. hydrogenoformans* using BLASTP with an E-value cutoff of 1e-5. Putative orthologs defined by mutual-best-hit methodology are present for only one third of those genes in *C. hydrogenoformans.* Among those missing genes are *spo0B* and *spo0F,* which encode the key components of the complex phosphorelay pathway in *B. subtilis* that channels various signals such as DNA damage, the ATP level, and cell density to the master switch protein Spo0A and therefore governs the cell's decision to enter sporulation. *C. hydrogenoformans* hence uses either a simplified version of this pathway or an alternative signal transduction pathway to sense the environmental or physiological stimuli. A large number of genes involved in the protective outer layer (cortex, coat, and exosporium) formation, spore germination, and small acid-soluble spore protein synthesis, among a few genes in various stages of spore development, are also missing. A similar, but slightly different, set of genes are missing in the other spore-forming *Clostridia* species as well [41]. Absence of those genes is more pronounced in non-spore-forming Firmicutes such as *Listeria* spp., *Staphylococcus* spp., and *Streptococcus* spp., as they lack all the sporulation-specific genes. When overlaid onto the phylogeny of Firmicutes (Figure 2), this observation can be explained by either multiple independent gene-loss events along branches leading to non-*Bacillus* species or by independent gene-gain events along branches leading to *Bacillus* and *Clostridia,* or by both. Whatever the history is of the sporulation evolution, the core set of sporulation genes shared by *Bacillus* and *Clostridia* might be close to a “minimal” sporulation set, as so far only these two groups have been found to be capable of producing endospores. Alternatively, some spore specific functions may be carried out by non orthologous genes in different species, which would prevent us from identifying them by this type of analysis.

### Strictly Dependent on CO?

Until very recently, *C. hydrogenoformans* was thought to be an autotroph strictly depending on CO for growth. An overview of the genome reveals features related to its autotrophic lifestyle. For example, it has lost the entire sugar phosphotransferase system and encodes no complete pathway for sugar compound degradation. However, many aspects of the gene repertoire are suggestive of heterotrophic capabilities. For example, among the transporters encoded in the genome are ones predicted to import diverse carbon compounds including formate, glycerol, lactate, C4-dicarboxylate (malate, fumarate, or succinate; the binding receptor for this has three paralogs in the genome), 2-keto-3-deoxygluconate, 2-oxoglutarate, and amino acids. In addition there is a diverse array of signal transduction pathways including chemotaxis not commonly found in the genomes of autotrophs (see below). Consistent with these observations, Henstra et al. recently showed that formate, lactate, and glycerol could be utilized as carbon source provided 9,10-anthraquinone-2,6-disulfonate was used as the electron acceptor [8]. Similarly, sulfite, thiosulfate, sulfur, nitrate, and fumarate were reduced with lactate as electron donor, although heterotrophic growth was relatively slow compared with cultures growing on pure CO [8]. It is not known what electron acceptors are likely to be coupled to these pathways in the isolation locale of *C. hydrogenoformans,* however it is clear that there is a more versatile complement of energy sources than initially concluded by Svetlichny et al. [2].

In terms of autotrophic lifestyle, although *C. hydrogenoformans* and *S. thermophilum* are close phylogenetically, they have gone separate ways in their lifestyles. *S. thermophilum* is an uncultivable thermophilic bacterium growing as part of a microbial consortium [18], while *C. hydrogenoformans* is a hot-spring autotroph that can survive efficiently on CO as its sole carbon and energy source. Accordingly, their metabolic capabilities are very different and only half of their proteomes are homologous. It is not clear why *S. thermophilum* is dependent on other microbes. Unlike other symbiotic microorganisms, no large-scale genome reductions have occurred in *S. thermophilum* [18]. On the other hand, *C. hydrogenoformans* has evolved to live preferably on CO, possibly by acquiring and/or expanding its complement of CODHs. As a result, it has lost many genes associated with a heterotrophic lifestyle, such as the phosphotransferase transporter system, and may be on the verge of becoming an obligate autotroph. Even though *C. hydrogenoformans* is more closely related to *S. thermophilum* than to *T. tengcongensis,* an anaerobic thermophile isolated also from freshwater hot springs [42], *C. hydrogenoformans* actually shares slightly less genes with *S. thermophilum* than with *T. tengcongensis.*

### Signal Transduction

*C. hydrogenoformans* is poised to respond to diverse environmental cues through a suite of signal transduction pathways and processes. The organism has 83 one-component regulators and 13 two-component systems (including two chemotaxis systems), which are average numbers for such a genome size [43] (Table S1). Many of the genes encoding these two-component systems are next to transporters, possibly being involved in regulation of solute uptake, while others are adjacent to oxidoreductases. *C. hydrogenoformans* also possesses an elaborate cascade of chemotaxis genes, including 11 chemoreceptors, and a complete set of flagellar genes, most located within a large cluster of about 70 genes (CHY0963–1033). Chemotaxis allows microbes to respond to environmental stimuli by swimming toward nutrients or away from toxic chemicals. Generally, a heavy commitment to chemotaxis is not a characteristic of autotrophic microorganisms [44], and it is possible that *C. hydrogenoformans* is responding to gradients of inorganic nutrients, or gases such as CO, O_2_, H_2_, or CO_2_.

Critical for sensing CO, two CooA homologs occur in the *C. hydrogenoformans* genome, both of which are encoded within operons containing *cooS* genes. CooA proteins are heme proteins that act as both sensors for CO as well as transcriptional regulators. They belong to the cyclic adenosine monophosphate receptor protein family and induce CO-related genes upon CO binding [45]. CHY1835, encoding CooA1, is at the beginning of the *R. rubrum*-like *coo* operon. CHY0083, encoding CooA2, is at the end of the operon possibly involved in NADPH generation from CO [1] (Figure 3).

*C. hydrogenoformans* lacks certain subfamilies of transcription factors that are present in its close *Clostridia* relatives, such as those utilizing the following helix-turn-helix domains: iron-dependent repressor DNA-binding domain, LacI, PadR, and DeoR (Pfam nomenclature). The genome does not encode any proteins of the LuxR family, which are usually abundant in both one-component (e.g., quorum-sensing regulators) and two-component systems.

The largest family of transcriptional regulators in *C. hydrogenoformans* is sigma-54- dependent activators. Eight such regulators comprise one-component systems (CHY0581, CHY0788, CHY1254, CHY1318, CHY1359, CHY1376, CHY1547, and CHY2091) and another one is a response regulator of the two-component system (CHY1855). Seven one-component sigma-54-dependent regulators have at least one PAS domain as a sensory module. PAS domains are known to often contain redox-responsive cofactors, such as FAD, FMN, and heme and serve as intracellular oxygen and redox sensors [46]. Overall, there are 18 PAS domains in *C. hydrogenoformans.* It is a very significant number compared to only two PAS domains in *Moorella thermoacetica* (similar genome size) and nine in *Desulfitobacterium hafniense* (a much larger genome). The most abundant sensory domain of bacterial signal transduction, the LysR substrate-binding domain, which binds small molecule ligands, is present only in six copies in *C. hydrogenoformans* (there are 36 copies in *D. hafniense*), re-enforcing the notion that redox sensing via PAS domains might be the most critical signal transduction event for this organism.

The most intriguing signal transduction protein in *C. hydrogenoformans* is the sigma-54-dependent transcriptional regulator that has an iron hydrogenase-like domain as a sensory module (CHY1547). This domain contains 4Fe-4S clusters and is predicted to use molecular hydrogen for the reduction of a variety of substrates. Its fusion with the sigma-54 activator and the DNA-binding HTH_8 domain in the CHY1547 protein strongly suggests that this is a unique regulator that activates gene expression in *C. hydrogenoformans* in response to hydrogen availability. Interestingly, it is located immediately upstream of a ten-gene cluster encoding a Ni/Fe hydrogenase (CHY1537–46). Iron hydrogenases similar to the one in CHY1547 can be identified in several bacterial genomes including *S. thermophilum, Dehalococcoides ethenogenes,* and some *Clostridia;* however, they are not associated with DNA-binding domains. The only organisms where we found a homologous sigma-54 activator are *M. thermoacetica, Geobacter metallireducens, G. sufurreducens,* and *Desulfuromonas acetoxidans.*

### Selenocysteine-Containing Proteins

*C. hydrogenoformans* possesses all known components of the selenocysteine (Sec) insertion machinery (CHY1803:SelA, CHY1802:SelB, CHY2058:SelD) and the Sec tRNA. A total of 12 selenocysteine-containing proteins (selenoproteins) were identified in *C. hydrogenoformans* genome by the Sec/Cys homology method (Table 3). For each of them, an mRNA stem-loop structure, the signature of the so-called Sec Insertion Sequence (SECIS) required for the Sec insertion, is present immediately downstream of the UGA codon. Although most of the identified selenoproteins are redox proteins, as has been shown for other bacteria and archaea [47], three are novel. Two are transporters (CHY0860, CHY0565), while the third is a methylated-DNA-protein-cysteine methyltransferase (CHY0809), a suicidal DNA repair protein that repairs alkylated guanine by transferring the alkyl group to the cysteine residue at its active site. It is striking that although this protein has been found in virtually every studied organism, only the one in *C. hydrogenoformans* has selenocysteine in place of cysteine at its active site. Therefore, this selenoprotein most likely evolved very recently, probably from a cysteine-containing protein. Similar patterns exist for the two selenocysteine-containing transporters, suggesting invention of new selenoproteins is an ongoing process in *C. hydrogenoformans.*

**Table 3:**
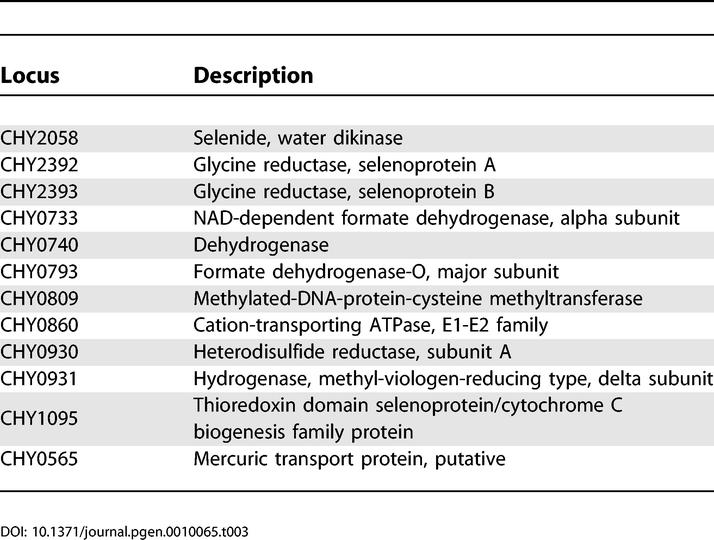
Selenoproteins Identified in *C. hydrogenoformans* Genome

### Translational Frameshifts

Analysis of the genome identified many potential cases of frameshifted genes. They are identified by having significant sequence similarity in two reading frames to a single homolog in another species. Examination of sequence traces suggests they are not sequencing errors. Some of these appear to be programmed frameshifts. Programmed frameshifting is a ubiquitous mechanism cells use to regulate translation or generate alternative protein products [48]. The frameshift in the gene *prfB* (CHY0163), encoding the peptide chain release factor 2, is a well-studied example of programmed frameshift that actually regulates its own translation [48].

However, many of the detected frameshifts appear to be the result of mutations from an ancestral un-frameshifted state. This is best exemplified by examination of the frameshift in the *cooS*-III gene (CHY1221), which as described above is predicted to encode one of the key components of the acetyl-CoA carbon fixation pathway. In cultures of another strain of this species (DSM 6008), a functional full-length (i.e., unframeshifted) version of this protein has been purified [24] and sequence comparisons of the gene from that strain with ours revealed many polymorphisms, including a deletion in our strain that gave rise to this frameshift (unpublished data). Studies of DSM 6008 show that in cultures grown in excess CO, the acetyl-CoA synthase (ACS, CHY1222) existed predominantly as monomer and only trace amount of CODH-III/ACS complex could be detected. On the other hand, when the CO supply was limited, CODH-III/ACS complex became the dominant form. It is plausible that CODH-III is not absolutely required for carbon fixation when the CO supply is high. Thus the frameshift and other mutations in *cooS-III* in Z-2901 may reflect the fact that it has been serially cultured in excess CO in the lab for many years. The putative lab-acquired mutations in Z-2901 are yet another reason to sequence type strains of species that have been directly acquired from culture collections and not submitted to extended laboratory culturing [49].

### Conclusion

Living solely on CO is not a simple feat and the fact that *C. hydrogenoformans* does it so well makes it a model organism for this unusual metabolism. Our analysis of the genome sequence, and phylogenomic comparisons with other species, provide insights into this species' specialized metabolism. Perhaps most striking is the presence of genes that apparently encode five distinct carbon monoxide dehydrogenase complexes. Analysis of the genome has also revealed many new perspectives on the biology and evolution of this species, for example, leading us to propose its reclassification, providing further evidence that it is not a strict autotroph and revealing a previously unknown ability to sporulate. The analysis reported here and the availibility of the complete genome sequence should catalyze future studies of this organism and the hydrogenogens as a whole.

## Materials and Methods

### Medium composition and cultivation.

*C. hydrogenoformans* Z-2901were cultivated under strictly anaerobic conditions in a basal carbonate-buffered medium composed as described [2]. However, 1.5 g l^−1^ NaHCO_3_, 0.2 g l^−1^ Na_2_S · 9 H_2_O, 0.1 g l^−1^ yeast extract, and 2 μmol l^−1^ NiCl_2_ were used instead of reported concentrations, and the Na_2_S concentration was lowered to 0.04 g l ^−1^. Butyl rubber-stoppered bottles of 120 ml contained 50 ml medium. Bottles were autoclaved for 25' at 121 °C. Gas phases were pressurized to 170 kPa and were composed of 20% CO_2_ and either 80% of N_2_, H_2_, or CO. Sporulation was induced by the addition of 0.01 mM MnCl_2_ to the medium and by a transient heat shock treatment (100 °C for 5 min).

### EM of *C. hydrogenoformans* endospore.

Samples were fixed with 5% glutaraldehyde for 2 h and 1% OsO_4_ for 4 h at 4 °C and then embedded in Epon-812. The thin sections were stained with uranyl acetate and lead citrate according to the method described by Miroshnichenko et al. [50]. The samples were observed and photographed using a JEOL JEM-1210 electron microscope.

### Genome sequencing.

Genomic DNA was isolated from exponential-phase cultures of *C. hydrogenoformans* Z-2901. This strain was acquired by Frank Robb from Vitali Svetlitchnyi (Bayreuth University, Germany) in 1995 after being serially grown in culture since its original isolation in 1990. Cloning, sequencing, assembly, and closure were performed as described [51,52]. The complete sequence has been assigned GenBank accession number CP000141 and is available at http://www.tigr.org.

### Annotation.

The gene prediction and annotation of the genome were done as previously described [51,52]. CDSs were identified by Glimmer [53]. Frameshifts or premature stop codons within CDSs were identified by comparison to other species and confirmed to be “authentic” by either their high quality sequencing reads or re-sequencing. Repetitive DNA sequences were identified using the REPUTER program [54].

### Comparative genomics.

To identify putative orthologs between two species, both of their proteomes were BLASTP searched against a local protein database of all complete genomes with an E-value cutoff of 1e-5. Species-specific duplications were identified and treated as one single gene (super-ortholog) for later comparison. Pair-wise mutual best-hits were then identified as putative orthologs.

### Genome tree construction.

Protein sequences of 31 housekeeping genes *(dnaG, frr, infC, nusA, pgk, pyrG, rplA, rplB, rplC, rplD, rplE, rplF, rplK, rplL, rplM, rplN, rplP, rplS, rplT, rpmA, rpoB, rpsB, rpsC, rpsE, rpsI, rpsJ*, *rpsK, rpsM, rpsS, smpB, tsf)* from genomes of interest were aligned to pre-defined HMM models and ambiguous regions were auto-trimmed according to an embedded mask. Concatenated alignments were then used to build a maximum likelihood tree using phyml [55].

### Phylogenetic profile analysis.

For each protein in *C. hydrogenoformans,* its presence or absence in every complete genome available at the time of this study was determined by asking whether a putative ortholog was present in that species (see above). Proteins were then grouped by their distribution patterns across species (bits of 1 and 0, 1 for presence and 0 for absence) using the CLUSTER program and the clusters were visualized using the TREEVIEW program (http://rana.lbl.gov/EisenSoftware.htm). Species were weighted by their closeness to each other to partially remove the phylogenetic component of the correlation [56].

### Identification of selenoproteins.

Each CDS of *C. hydrogenoformans* that ends with stop codon TGA was extended to the next stop codon TAA or TAG. It was then searched with BLASTP against the nraa database. A protein with a TGA codon pairing with a conserved Cys site was identified as a putative selenoprotein. The secondary structure of the mRNA immediately downstream of the TGA codon was also checked using MFOLD [57] to look for a possible stem-loop structure.

## Supporting Information

Table S1 Regulatory Genes in *Clostridia* Species (22 KB DOC)

## Abbreviations

CDS: protein coding sequence
CO: carbon monoxide
CODH: carbon monoxide dehydrogenase

## Note Added in Proof

It has come to our attention that a complementary comparison of sporulation genes in various Firmicutes was published in 2004 [58]. This study identified homologs of known sporulation genes in Firmicutes by experimental methods and genome analysis. The authors then used these results to study the evolution of sporulation and known sporulation genes.
