# Correction: Life in Hot Carbon Monoxide: The Complete Genome Sequence of Carboxydothermus hydrogenoformans Z-2901

**DOI:** 10.1371/journal.pgen.0020060

**Published:** 2006-04-28

**Authors:** Martin Wu, Qinghu Ren, A. Scott Durkin, Sean C Daugherty, Lauren M Brinkac, Robert J Dodson, Ramana Madupu, Steven A Sullivan, James F Kolonay, Daniel H Haft, William C Nelson, Luke J Tallon, Kristine M Jones, Luke E Ulrich, Juan M Gonzalez, Igor B Zhulin, Frank T Robb, Jonathan A Eisen

DOI: 10.1371/journal.pgen.0010065

In *PLoS Genetics,* volume 1, issue 5:

The list of contributing authors does not include Daniel H. Haft, whose name should have appeared between James F. Kolonay and William C. Nelson. He participated in analysis of the data.

