# Supplementary material for: Life in Hot Carbon Monoxide: The Complete Genome Sequence of Carboxydothermus hydrogenoformans Z-2901
Source: PLoS Genet. 2005 Nov 25;1(5):e65. doi: 10.1371/journal.pgen.0010065 (PMC1287953; doi:10.1371/journal.pgen.0010065)
Supplement: Supplementary file 1 [file pgen.0010065.st001.doc]

Table S1. Number of regulatory genes in *Clostridia* species

| Species | Genome size (Mb) | One-component | Two-component |
| --- | --- | --- | --- |
| *Carboxydothermus hydrogenoformans* | 2.40 | 83 | 13 |
| *Moorella thermoacetica* | 2.63 | 85 | 14 |
| *Thermoanaerobacter tengcongensis* | 2.69 | 79 | 16 |
| *Clostridium tetani* | 2.87 | 88 | 30 |
| *Clostridium perfringens* | 3.09 | 96 | 20 |
| *Symbiobacterium thermophilum* | 3.57 | 89 | 35 |
| *Clostridium thermocellum* | 3.74 | 88 | 33 |
| *Clostridium acetobutylicum* | 4.13 | 176 | 43 |
| *Desulfitobacterium hafniense* | 6.96 | 245 | 80 |
